# High-threshold motor unit firing reflects force recovery following a bout of damaging eccentric exercise

**DOI:** 10.1371/journal.pone.0195051

**Published:** 2018-04-09

**Authors:** Lewis J. Macgregor, Angus M. Hunter

**Affiliations:** Faculty of Health Sciences and Sport, University of Stirling, Stirling, Scotland; University of Tennessee Health Science Center College of Graduate Health Sciences, UNITED STATES

## Abstract

Exercise-induced muscle damage (EIMD) is associated with impaired muscle function and reduced neuromuscular recruitment. However, motor unit firing behaviour throughout the recovery period is unclear. EIMD impairment of maximal voluntary force (MVC) will, in part, be caused by reduced high-threshold motor unit firing, which will subsequently increase to recover MVC. Fourteen healthy active males completed a bout of eccentric exercise on the knee extensors, with measurements of MVC, rate of torque development and surface electromyography performed pre-exercise and 2, 3, 7 and 14 days post-exercise, on both damaged and control limb. EIMD was associated with decreased MVC (235.2 ± 49.3 Nm vs. 161.3 ± 52.5 Nm; p <0.001) and rate of torque development (495.7 ± 136.9 Nm.s-1 vs. 163.4 ± 163.7 Nm.s-1; p <0.001) 48h post-exercise. Mean motor unit firing rate was reduced (16.4 ± 2.2 Hz vs. 12.6 ± 1.7 Hz; p <0.01) in high-threshold motor units only, 48h post-exercise, and common drive was elevated (0.36 ± 0.027 vs. 0.56 ± 0.032; p< 0.001) 48h post-exercise. The firing rate of high-threshold motor units was reduced in parallel with impaired muscle function, whilst early recruited motor units remained unaltered. Common drive of motor units increased in offset to the firing rate impairment. These alterations correlated with the recovery of force decrement, but not of pain elevation. This study provides fresh insight into the central mechanisms associated with EIMD recovery, relative to muscle function. These findings may in turn lead to development of novel management and preventative procedures.

## Introduction

Exercise-induced muscle damage (EIMD) impairs force, and is usually accompanied by delayed onset muscle soreness [1] and inflammation [2,3]. Furthermore, these symptoms may, in turn, feedback to the central nervous system, contributing towards further force impairment [4,5]. Although interconnected, these symptoms have been shown to recover at varying rates, with force deficits having been reported up to 4 [6] and 6 days [7] post-EIMD, while, in the same studies, muscle soreness was seen to recover by 3 days. While mechanisms of EIMD have been widely discussed [8], recovery remains comparatively poorly understood [9]. Much of the research into exercise recovery is focused around the impact of peripheral alterations, while the influence of central factors demands further study [10,11]. Understanding the underlying mechanisms behind the recovery process can have particularly valuable implications among groups known to suffer from impaired recovery, such as elderly and clinical populations [12,13].

Altered afferent signalling to the central nervous system may modify neuromuscular recruitment strategy, although it has been demonstrated that both muscle spindle [14] and Golgi tendon organ function [15] remain undamaged following eccentric exercise. Alternatively, nociceptor sensitisation has been shown to elevate III/IV afferent signalling [16], which can lead to impaired muscle performance [17]. Despite this, the recovery time course of these alterations and how they link to functional symptoms of EIMD remains unclear. Following EIMD, changes in motor unit recruitment threshold and discharge rate have been reported [18], as well as motor unit conduction velocity [19] and synchronization [20], all occurring within 24h post-EIMD; indeed, Piitulainen et al (2010) [19] reported increased motor unit firing 2h post-exercise only. However, motor unit synchronization has been reported to remain elevated 7d post-exercise, despite recovery of MVC [20]. Given that EIMD impairs muscular force production for up to ten days following damaging exercise [7,21–23], altered neural control strategy, during muscle contraction, is likely, until recovery is achieved.

Methodologies for measuring neural control strategy have included the use of indwelling electrodes to record up to ~10 individual motor units, during low intensity contractions. In order to decipher details of motor unit behaviour from larger motor unit pools, constituent motor unit action potential trains (MUAP) can now be extracted from surface electromyographical (sEMG) signals, using either high density EMG [24,25] or precision decomposition EMG (dEMG) [26–29]. dEMG has been designed to investigate the behaviour of a sample of motor units, representative of the active motor unit pool [26,30]. Motor unit discharge onto the sarcolemma is captured from a single multi-channel sEMG electrode array sensor, recorded during voluntary contraction at any given load. The dEMG system allows assessments of motor unit firing properties during contractions up to levels close to MVC [28] and facilitates evaluation of different motor units within the recruited pool, based on their recruitment threshold [31]. Given that motor unit recruitment in vastus lateralis occurs at up to 95% of MVC [32], by facilitating measurement of motor unit properties at higher contraction intensities, we are able to investigate motor unit behaviour within a wider sample of motor units, with differing firing characteristics [33], than can be activated using indwelling EMG techniques.

High force producing type II (fast oxidative/ glycolytic) muscle fibres are more susceptible to EIMD than type I (slow oxidative) fibres [34,35]. It is therefore likely there will also be preferential impairment of specific motor units. Therefore, when applying the order of size recruitment principle [36] it seems plausible that later recruited motor units would become affected, although to date this has not been established. Recently, it was shown that eccentric exercise caused an alteration in the relationship between motor unit firing rate (MUFR) and recruitment threshold [37], this suggests that EIMD might disrupt the later-recruited motor units, which in all likelihood are associated with type II muscle fibres [36]. However, these later-recruited motor units have not been investigated in isolation from the entire active motor unit pool, either during EIMD, or during subsequent recovery. Understanding neural control strategies associated with EIMD recovery is essential to allow optimal management of impaired muscles.

While previous studies [19,20,38,39] have investigated EIMD in the highly susceptible elbow flexors, knee extensor muscles are more functionally relevant for daily mobility. Furthermore, to our knowledge, no study has established the neural code for controlling force production during complete EIMD recovery. Therefore, the aim of the present study was to investigate the time course recovery of EIMD-associated alterations in MUFR and common drive in vastus lateralis, using dEMG. Specifically, MUFR will be analysed by dividing the motor unit pool into early-recruited, mid-recruited and later recruited units, as previously reported [40]. This will enable us to establish the firing behaviour of high-threshold motor units, in isolation from the entire motor unit pool. It was hypothesized that the firing rate would be altered in high-threshold motor units only, and that common drive to the motor unit pool would be similarly affected following EIMD, and that these alterations would follow a similar time course recovery to impairments in MVC.

## Materials and methods

### Subjects

Fourteen healthy, recreationally active, male participants with no history of neuromuscular or musculoskeletal disorders were recruited (age 25.4 ± 5.4y, height 1.8 ± 0.1m, body mass 79.0 ± 12.0Kg and knee extension strength (MVC) at baseline 233.2 ± 47.7Nm). Volunteers provided written consent, having been informed of any potential risks involved in their participation, the study was approved by the local Research Ethics Committee (SSEC). All procedures performed in studies involving human participants were in accordance with the ethical standards of the 1964 Helsinki declaration and its later amendments. All participants were deemed to be unaccustomed to eccentric resistance exercise, for at least the six months prior to their participation in the study. Participants refrained from: 1) any unaccustomed physical activity for the duration of the trial and 2) any strenuous exertion for at least 24h prior to each testing session.

### Study design

Following full familiarisation of the testing procedures, participants reported to the laboratory on five occasions, over a 14 day period. Food intake was recorded for 3 consecutive days, prior to beginning the trial. Participants reported to the laboratory following an overnight fast. Baseline measures were recorded for knee extensor muscle soreness, before isometric maximal voluntary contraction (MVC) and neuromuscular assessments were performed using an isokinetic dynamometer (Biodex System 3, Medical Systems, New York, USA). In all cases, measurements were carried out for the non-dominant (control) leg prior to the dominant (intervention) leg. The order of measurements was consistent across all trials.

### Protocol

Participants were coupled to the isokinetic dynamometer for assessment of MVC, neuromuscular measures and muscle soreness as well as for performing eccentric contractions to induce EIMD. The lateral femoral epicondyle of the testing leg was visually aligned with the axis of rotation of the dynamometer, and seat positions were adjusted to suit each individual participant’s anthropometric characteristics. In accordance with the manufacturers’ instructions straps (across the chest, pelvis and resting leg) were used to secure the participant in the required position, and to reduce mechanical assistance from other body parts. During contractions participants were instructed to cross their arms in front of their chest. The final positioning of each participant was recorded on the initial visit and replicated throughout the experimental period, to ensure constancy.

Participants rated perceived muscle soreness while positioned in the isokinetic dynamometer. Soreness was measured while the knee was fully extended (joint angle of 0^o^). Pressure was applied (1kg/cm) to the midpoint on the lateral and transverse planes of the Quadriceps Femoris, using a custom built, spring loaded algometer. Participants rated their level of soreness using a 200mm visual analogue scale (VAS) which ranged from ‘no pain’ at the extreme left to ‘most pain imaginable’ at the extreme right [41]. The two ends of the VAS were anchored by perpendicular lines, but there were no increments between the end markers. Participants were instructed to mark a point along the line which represented the perceived soreness felt as pressure was applied to the muscle. For each measurement a fresh scale was used, with no reference to previous measurements. Muscle soreness was quantified by measuring the distance (to the nearest 0.1cm) from the left anchor point to the point marked by the individual. During pre-exercise testing muscle soreness of the dominant leg was rated twice, once before baseline measures and once immediately following the cycling warm-up, to ensure that not muscle soreness occurred during the warm up.

With the participant secured in the dynamometer gravitational corrections were performed, in accordance with existing recommendations [42], in order to account for the effect of limb weight on torque measurements. A knee joint angle of 60^o^ was set and the limb was secured by a Velcro strap proximal to the medial malleolus. The angle of 60^o^ was chosen, as it lies within the well-established range of reported optimal knee joint angles, for peak isometric torque production [43]. Participants performed a standard submaximal warm-up, consisting of two sets of 3 x 5s isometric contractions; with 30s rest between repetitions and 60s recovery between sets. For the first set participants contracted at an intensity perceived to be 50% of maximum effort; for the second set the intensity of contraction was 75% of perceived maximum [7,44] visual feedback was available on a monitor positioned in front of the dynamometer seat, as an output guide.

Immediately following isometric warm up, participants performed 3 x 5s isometric maximal voluntary contractions (MVC). Participants were required to react to an audio prompt and were instructed to exert as much force as possible, as quickly as possible, in response to the prompt. The gap between prompts was randomized, such that participants could not anticipate their next contraction; in this way, an accurate contraction onset could be determined [45], allowing rate of torque development (RTD) to be calculated. The contraction containing the highest peak torque was designated MVC. From this contraction RTD was calculated over 0-300ms from the onset of contraction (± 2SD from baseline) using MATLAB version 7.11.0.584 (R2010b) software (The MathWorks, Inc.). Participants were instructed not to hold back any effort for subsequent contractions. The same investigator provided standardized verbal commands and encouragement, to assist the participants in achieving maximal effort for every contraction; coefficient of variation was ensured to be lower than 5% across sets of 3 contractions. Impaired muscle force, in combination with impaired velocity (RTD), is considered the most appropriate indirect measure of EIMD [46].

**Fig 1:**
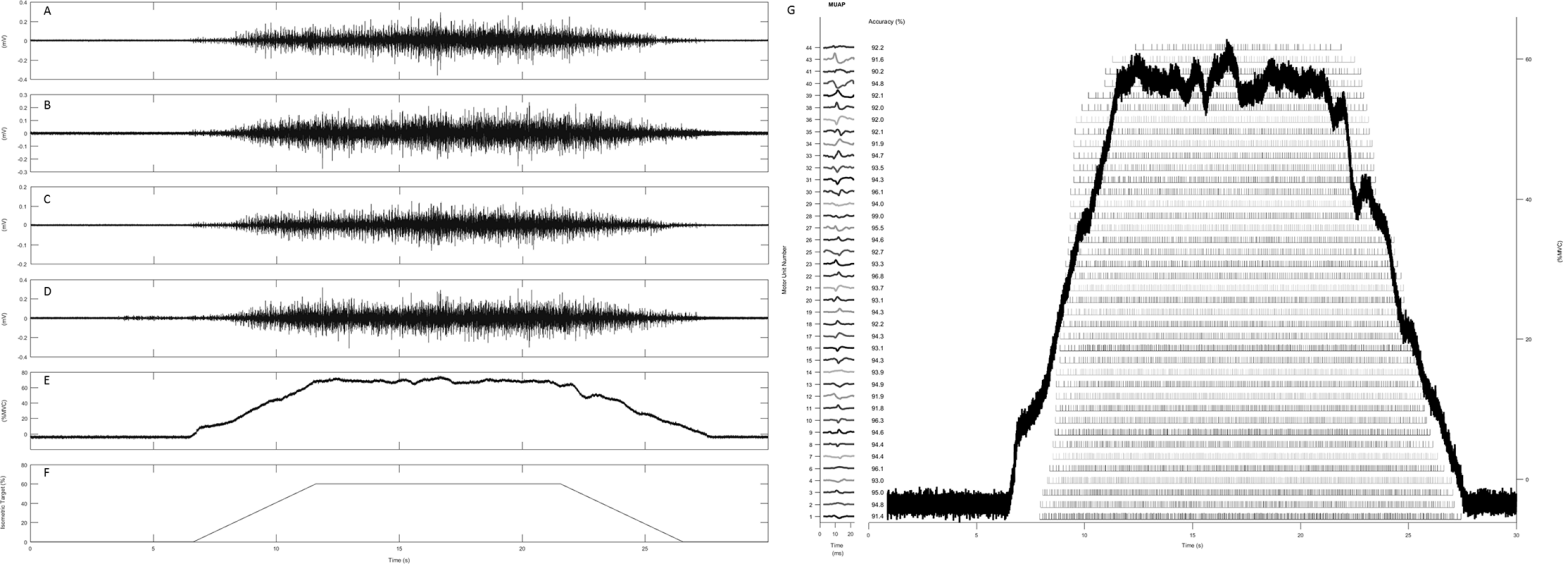
Example of raw sEMG captured concurrently on 4 channels (**A—D**), force output (Nm) and target (% of MVC) are also shown (in **E** and **F**, respectively). **G** Example of firing rate bar plot from one participant (40 motor units), vertical bars represent the firings of each motor unit. The force output trace is superimposed. Action potential shapes associated with each identified motor unit, and the results of Decompose-Synthesize-Decompose-Compare accuracy tests (%) are presented along the left vertical axis (motor units number 5, 24, 37 and 42 are absent, as they did not achieve the required accuracy %).

Following determination of baseline MVC, for each leg, subjects performed a submaximal isometric muscle action following a trapezoidal template (Fig 1E and 1F). Participants linearly increased the magnitude of isometric contraction, tracing the shape of the template, from 0–60% (of baseline MVC) for 6s, at a rate of ~10%^.^s^-1^; the contraction level was held steady at 60% for 10s, then linearly decreased from 60% - 0 at the same rate as above. Participants were instructed to completely relax the knee extensors at the start and end of each action; visual inspection of the quiescent portions before and after the signal confirmed that vastus lateralis was relaxed during these periods, and that no torque was generated. The template and output feedback trace were visible on a monitor positioned directly in front of the dynamometer. Participants were required to follow the template as closely as possible with their output trace. This contraction provided a stationary signal, sufficiently long to allow reliable decomposition of sEMG. The value of 60% of MVC was chosen, as studies have previously demonstrated force decrements of ≤40% following EIMD in knee extensors. However cross-correlation analysis of single motor units has typically been conducted using target forces <30% of MVC [47], limiting findings to motor units recruited at these lower forces. As EIMD predominantly affects higher force producing (type II) muscle fibres, it was critical to investigate MUFR responses at as high an isometric target force as possible, to permit study of a larger range of motor units.

A surface array dEMG sensor (Delsys, Inc., Boston, Massachusetts) was used to detect bipolar surface EMG signals, on four separate channels (Fig 1A–1D), from the vastus lateralis of each leg in turn, during isometric MVCs and submaximal trapezoid contractions. The sensor consisted of five cylindrical pin electrodes, each 0.5mm in diameter, protruding from the housing (2x3cm). The pins are blunted, such that they make an indentation when pressed against the skin, but do not puncture the surface. Four of the five pins are arranged at the corners of a 5x5mm square; the fifth (reference) pin is in the centre of the square, equidistant from each of the other four, such that the inter-electrode distance is 3.6mm [28]. Before placement, the skin over the distal region of the muscle was prepared by carefully shaving and then cleansing with rubbing alcohol, the skin was then abraded in accordance with SENIAM recommendations [48]. The sensor was first cleaned with rubbing alcohol, before fixing to the prepared skin with adhesive tape. The sensor was located over the belly of the vastus lateralis—25% of the distance from the Gerdy prominence to the AIS [49]. A reference electrode (HE-R, Dermatrode, American Imex, Irvine) was affixed to the patella; if it was deemed necessary, the investigator also shaved the skin over the patella before attaching the reference electrode.

Visual inspection of the signal, on all four channels, was carried out, prior to recording, to ensure that excessive background noise and artifact were not present; in accordance with the manufacturers guidelines, baseline noise should not exceed 10μV. Signal to noise ratio is calculated within the acquisition software, according to the formula: 20log(S_RMS_/N_RMS_) [where S = EMG signal and N = baseline noise]. All analog EMG signals were low-pass (fourth-order Butterworth, 24 dB/octave slope, 1750-HZ cut-off) and high-pass (second-order Butterworth, 12 dB/octave slope, 20-HZ cut-off) filtered prior to sampling at a rate of 20,000 Hz [31,50]. The four separate filtered EMG signals from the array were entered into the Precision Decomposition III (PD III) algorithm and decomposed into constituent motor unit action potential trains (EMGworks® 4.0 Analysis software, Delsys, Boston, USA). Precision Decomposition techniques were originally described by Adam & De Luca (2005) [26], having been in development since the 1970s. The technique has subsequently been refined by Nawab et al (2010) [28]. PD III uses artificial intelligence to identify action potentials and assign them to individual motor units; reliability and validity have been described previously in a variety of contraction conditions, including high-intensity contractions up to 80% and 100% MVC [31,50–53]. This technique was specifically developed for decomposing surface EMG signals into their constituent MUAPs, and has previously been utilised to examine altered neural strategies in patients affected by stroke [54–56] and poliomyelitis [57]. The resulting output from the decomposition algorithm contains the firing instances, in pulses per second (pps), for each motor unit (Fig 1G).

To assess the accuracy of the decomposed signal a Decompose-Synthesize-Decompose-Compare test, as described by De Luca & Hostage (2010) [51] was performed. This test is currently considered the most suitable way of validating the decomposition of the surface EMG signal [29,58,59]. On average, PD III decomposition, according to the Decompose-Synthesize-Decompose-Compare test, showed accuracy ≥94.1 ± 1.8%. Full accuracy data are provided in Table 1. The mean firing rate, for each active motor unit, can then be calculated and plotted as a function of time. Mean firing rate curves were smoothed using a Hanning window; in this case all curves were filtered using a 600ms Hanning window, as recommended by the Software manufacturer. For analysis a long enough portion of the mean firing rate curves was needed to allow fluctuations in firing rate to be analyzed, however excessively long portions are not desirable, as the period should include minimal fluctuations in force or EMG RMS. A 3s portion has previously been deemed, by our group, to be appropriate [40]. The 3s period at the distal end of the contractions steady-phase was found to be the region of greatest reliability.

**Table 1:** Results of Decompose-Synthesize-Decompose-Compare accuracy test. Values are mean ± SD, n = 14.

Day		0	2	3	7	14
**Control**	**Mean (%)**	**95.1**	**95.7**	**95.7**	**95.1**	**94.1**
	**± SD**	**2.6**	**2.5**	**2.3**	**2.7**	**1.8**
**Damage**	**Mean (%)**	**96.1**	**96.3**	**96.3**	**94.3**	**95.2**
	**± SD**	**2.3**	**2.1**	**2.1**	**4.8**	**2.3**

Recruitment threshold for each motor unit was calculated as the relative force (% of MVC) compared with the mean firing rate, as previously described [60]. MUAPs were next separated into three groups, containing equal numbers of motor units, (where MUAPs could not be equally divided by three, the third group contained any additional motor units), such that MUAPs could be isolated into tertiles containing either early recruited, mid-recruited or later recruited motor units [40]. This concept is a similar to that described elsewhere [33,61], defining low and high-threshold motor units by comparing motor units recruited above vs. below a predetermined threshold (% of MVC). However, allocating motor units into tertiles provides greater separation between early- and later-recruited motor units. The mean relative recruitment thresholds (% of MVC) of motor units allocated to each tertile are shown in Table 2. The level of common drive was quantified by performing cross-correlation analysis of all of the mean firing rate curves during the constant firing rate portions of the curves, using the formula:

$$(f*g)[n]≝\sum_{m=-\infty }^{+\infty }f^{*}[m]g[n+m]$$

**Table 2:** Mean ± SD force (% of MVC) at which early (T1), mid (T2) and late (T3) motor units were recruited during the submaximal isometric trapezoidal contraction.

	Day			0	2	3	7	14
**Control**	**Recruitment threshold (%)**	**Mean ± SD**	**T1**	**11.9 ± 0.7**	**15.1 ± 2.8**	**8.0 ± 1.4**	**6.8 ± 1.1**	**12.2 ± 2.3**
			**T2**	**14.8 ± 0.6**	**22.4 ± 1.8**	**12.8 ± 1.8**	**10.3 ± 0.9**	**19.3 ± 1.5**
			**T3**	**19.9 ± 4.4**	**32.7 ± 5.7**	**24.3 ± 8.2**	**19.9 ± 6.9**	**26.4 ± 5.2**
**Damage**	**Recruitment threshold (%)**	**Mean ± SD**	**T1**	**13.1 ± 2.6**	**24.9 ± 3.2**	**17.3 ± 1.1**	**10.9 ± 3.3**	**16.4 ± 3.9**
			**T2**	**19.6 ± 2.7**	**28.9 ± 0.9**	**21.5 ± 1.7**	**18.2 ± 1.9**	**23.5 ± 1.7**
			**T3**	**28.7 ± 5.5**	**35.6 ± 4.6**	**28.5 ± 5.0**	**32.3 ± 8.8**	**32.5 ± 6.9**

Common drive is calculated from mean motor unit firing rate data and represents simultaneous fluctuations in firing rate between pairs of motor units [62].The same 3s portion of the isometric contraction was analyzed for common drive as for mean firing rate. All possible combinations of motor units were cross-correlated with one another [39]. The peak cross-correlation coefficient and time lag were calculated from each cross-correlation to determine common drive.

### Eccentric exercise

Following baseline measurements on Day 0, subjects used their dominant leg to perform an eccentric exercise protocol, as outlined below, designed to induce temporary muscle damage. Repeat measures of all baseline characteristics were then taken on Days +2, +3, +7 and +14. The eccentric exercise was performed on one day only. During familiarization a maximum of one full set of twelve eccentric contractions was practiced.

Prior to the eccentric exercise participants performed a brief warm-up, consisting of cycling for 5min at a cadence of 70rpm with power output of 50w (Lode Excalibur Sport V2 electrically-braked cycle ergometer, Lode BV, Groningen, Netherlands), to our knowledge no evidence has been reported for this type of warm-up to cause muscle damage in healthy males. Immediately following the warm-up, participants were secured in the isokinetic dynamometer, (as described above) and measurements of muscle soreness were repeated, exactly as before, to ensure that no significant muscle soreness had resulted from the baseline measures and warm-up cycling.

Participants then performed sets of twelve maximal eccentric contractions, until exhaustion (i.e. failure to complete all twelve repetitions within a set); a minimum of 120 seconds recovery was permitted between each set. The range of movement of these contractions was 90^o^, participants were instructed to provide maximum resistance from knee extension angle 20^o^ to 110^o^ (full extension being 0^o^). The velocity of contraction was 60^o.^sec^-1^. Each eccentric contraction was followed by a passive return to start angle, at a velocity of 180^o.^sec^-1^, such that each set lasted for 24 seconds, with participants actively contracting for 75% of that time. This protocol, adapted from a number of previously published studies [63,64] is designed to maximize eccentric workload whilst concomitantly minimizing concentric work and metabolic demand. Subjects were verbally encouraged to generate maximum force during each eccentric contraction, throughout the whole range of movement. Participants were instructed to drink sufficient water following the eccentric exercise protocol to avoid a possible risk of acute renal failure due to rhabdomyolysis [46], but they were instructed to abstain from any therapeutic treatments, designed to ameliorate the symptoms of EIMD, prior to and throughout the trial period. Therapies to avoid included, but were not restricted to: whole-body vibration, massage, cryotherapy, non-steroidal anti-inflammatory drugs and branch-chained amino acids [8,65,66].

### Statistical analysis

MVC, RTD muscle soreness, mean motor unit firing rate of each tertile and common drive were tested for normality using Ryan-Joiner test, then analyzed using two-way (group, 2 x time, 5) repeated measures analysis of variance (ANOVA) with Tukey *post hoc* analysis performed where appropriate (Minitab 16 statistical software, Minitab Ltd., Coventry, UK). Data are presented throughout as mean ± standard deviation (SD), with statistical significance set at *P* < 0.05. Where significant effects were observed, Cohen’s *d* effect sizes (ES) were calculated by: Cohen's *d* = *Mean*_1_—*Mean*_2_ / SD_pooled_, where SD_pooled_ = √[(SD _1_^2^+ SD _2_^2^) / 2], and 95% lower and upper confidence intervals (CI) were established relative to ES. ES were interpreted as < 0.2 = trivial, 0.2–0.5 = small, 0.5–0.8 = moderate, > 0.8 = large [67].

## Results

**Fig 2:**
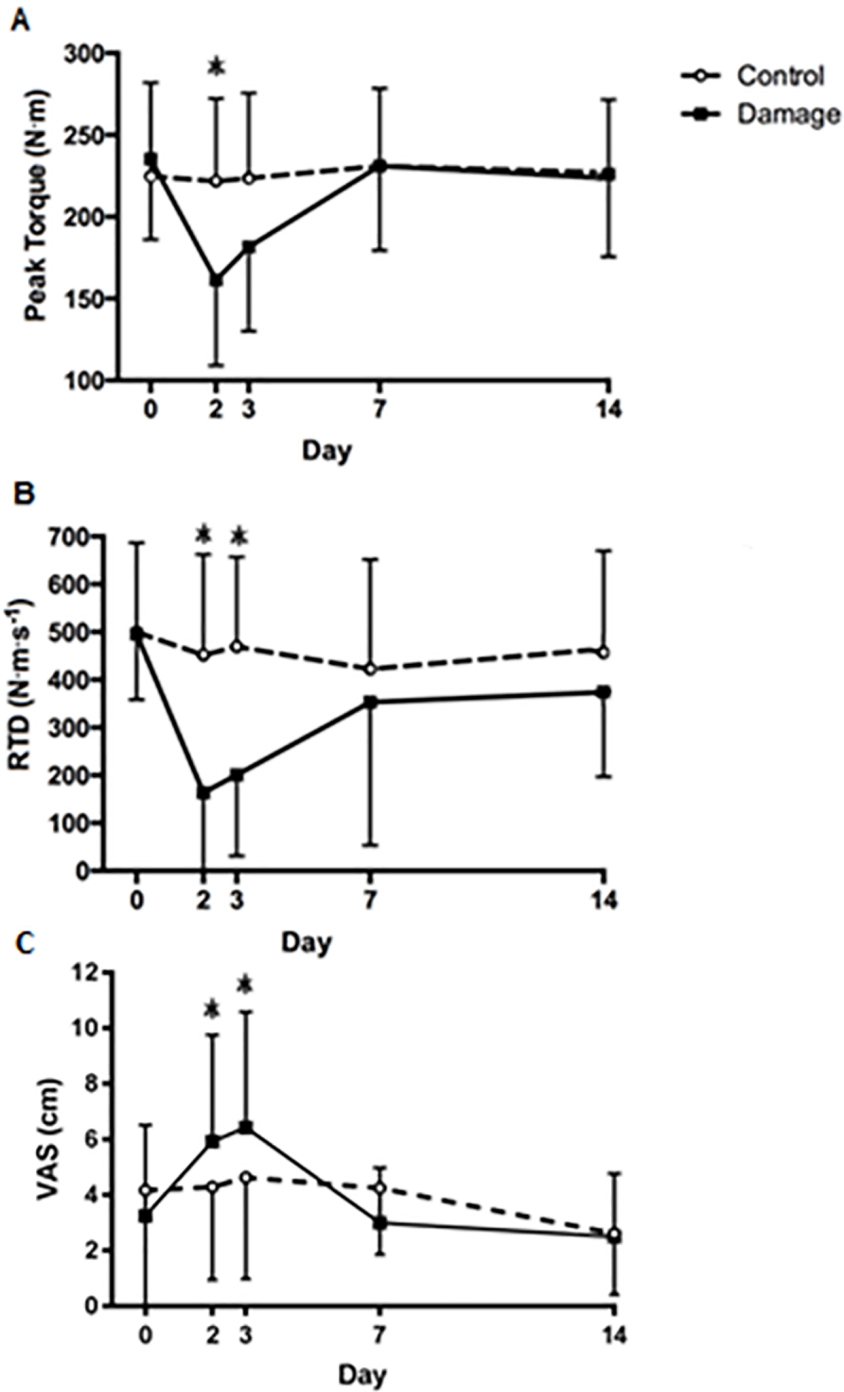
**A**) Maximal isometric voluntary contraction (MVC) of the exercised and control knee extensors. **B**) Rate of torque development (RTD) of the exercised and control knee extensors. **C**) Muscle soreness measured using visual analogue scale (VAS). Values are mean + SD, n = 14. * Significantly higher than baseline in the exercised leg, p <0.01.

EIMD was associated with significantly reduced MVC (*F*_(4,13)_ = 11.77, p <0.001) (Fig 2A) and showed significant interaction effects (*F*_(4,13)_ = 18.49, p <0.001) with the control leg; post hoc testing revealed significantly reduced MVC by 31.4% in the exercised leg at 48h compared to baseline (CI [0.58 to 2.24] ES = 1.45, p <0.01) which had fully recovered by day 7. RTD responded similarly by showing a significant (*F*_(4,13)_ = 9. 96, p <0.001) reduction of 67.04% in the exercised leg post-EIMD, with significant interaction effects (F_(4,13)_ = 9.49, p <0.001); post hoc testing revealed significantly reduced RTD compared to baseline in the exercised leg at 48 and 72h with peak reduction at 48h (CI [1.21 to 3.07] ES = 2.20, p < 0.01) which had recovered by day 7 (Fig 2B). Muscle soreness was significantly greater (*F*_(4,13)_ = 5.35, p <0.01) in the exercised leg following EIMD, post hoc testing revealed a main effect of time at 48 and 72h with peak elevation at 72h (CI [0.05 to 1.60] ES = 0.85 p <0.01) (Fig 2C).

**Fig 3:**
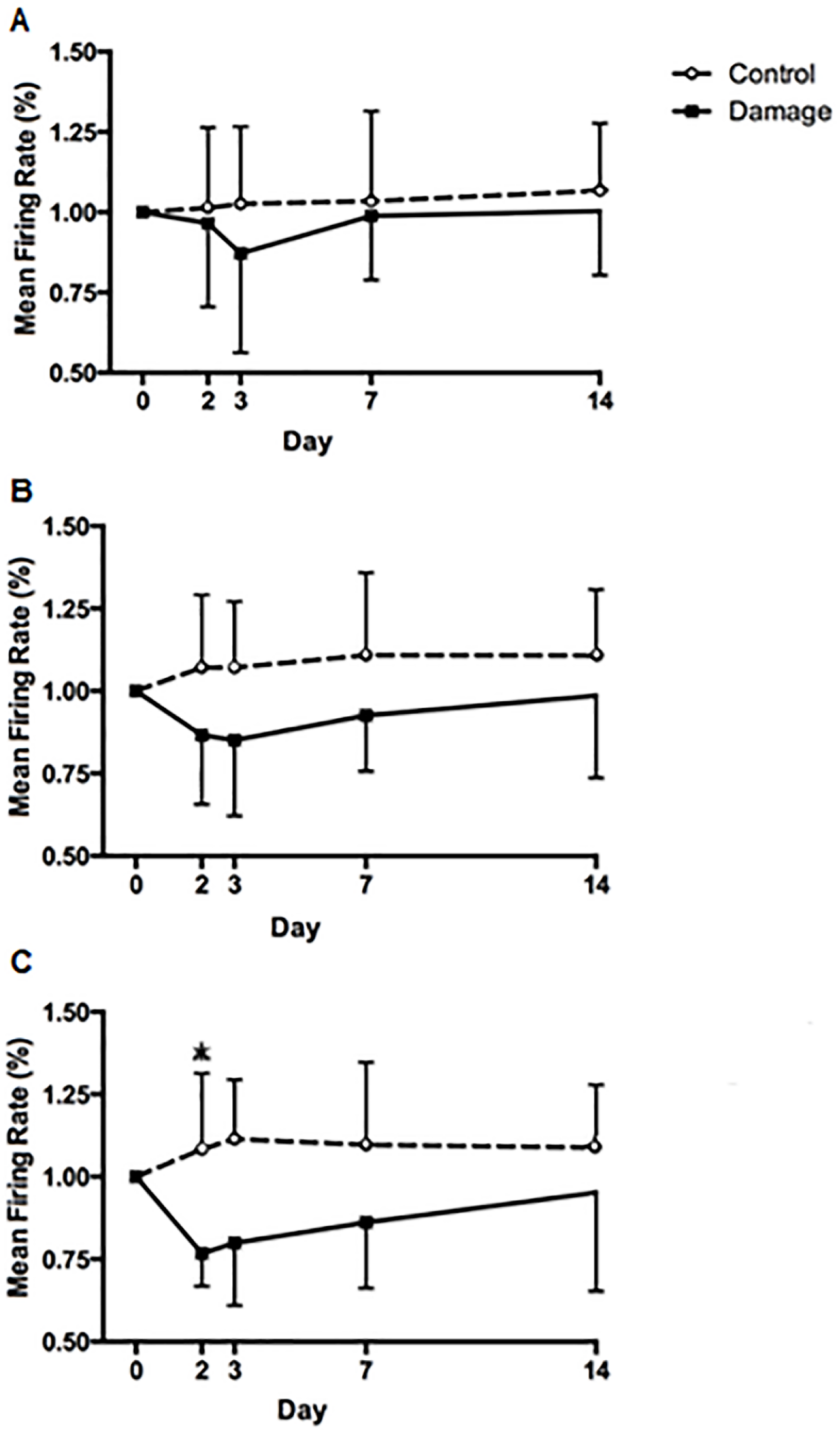
Mean motor unit firing rates of the exercised and control knee extensors. **A**) Early recruited motor units, **B**) mid recruited motor units and **C**) late recruited motor units. Values are % change + SD, n = 14. * Significantly lower than baseline in the exercised leg, p <0.05.

**Fig 4:**
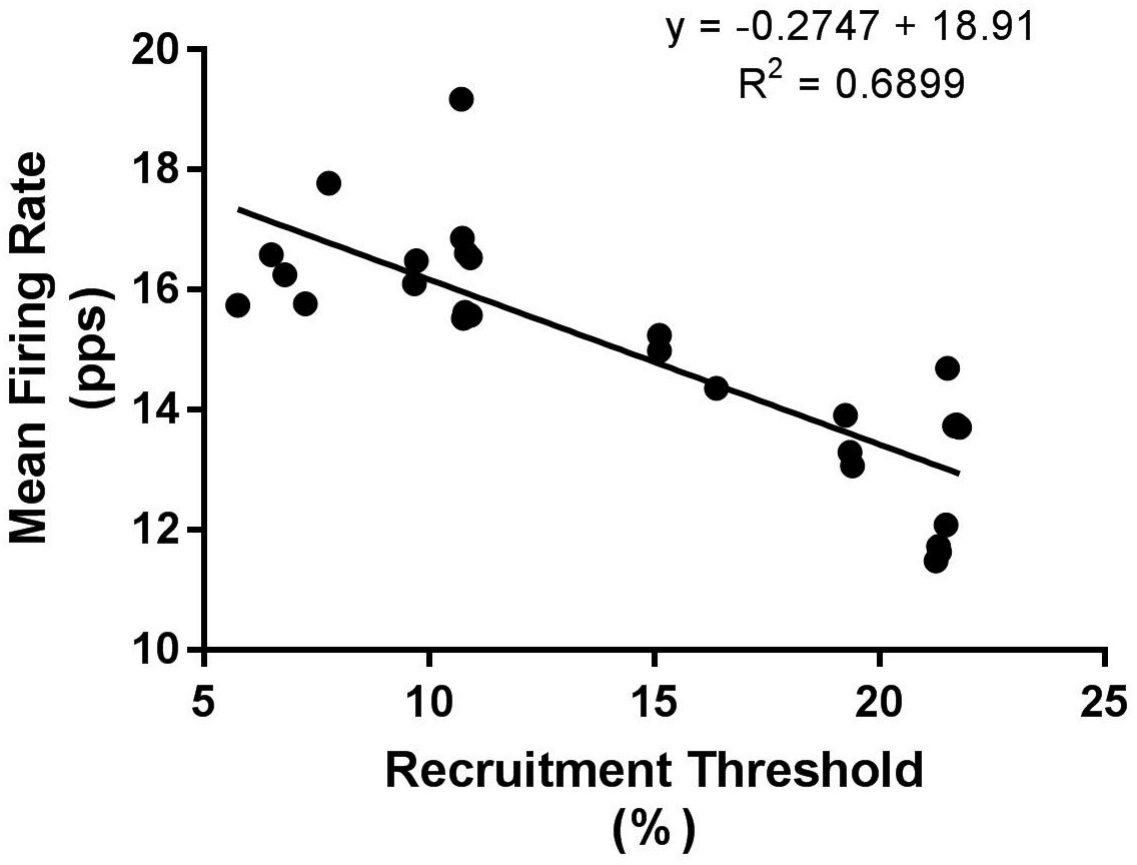
Example linear regression line for the relationship between motor unit mean firing rate and recruitment threshold for a single subject.

No differences existed in the relationship between average MUFR and the recruitment threshold (Table 3). However, higher-threshold motor units, in the third tertile fired significantly (*F*_(4,13)_ = 4.81, p <0.01) slower following EIMD demonstrating a significant interaction (*F*_(4,13)_ = 4.81, p <0.01) with the control leg and post hoc testing showing a significant decline at 48h (from 16.4 ± 2.1pps to 12.6 ± 1.7pps) (CI [1.01 to 2.79] ES = 1.96, p <0.05) which had returned to baseline levels after 72h (13.1 ± 2.37pps) (Fig 3C). The mid recruited motor units as shown by the second tertile demonstrated a tendency (*F*_(4,13)_ = 2.16, p = 0.093) towards lower mean firing rates in the exercised leg post-EIMD (Fig 3B). The mean firing rate of early recruited motor units, within the first tertile, was not significantly different (*F*_(4,13)_ = 1.19, p >0.05) between days or groups (Fig 3A). An example of regression analysis of recruitment threshold (% of MVC) against mean firing rate is presented in Fig 4.

**Table 3:** Mean linear slope coefficients (pps^(*)^/%MVC) and y-intercept (pps) relationships between average firing rate and recruitment threshold of motor units. Values are mean ± SD, n = 14.

	Day		0	2	3	7	14
**Control**	**Slope coefficient**	**Mean**	**0.51**	**0.67**	**0.55**	**0.58**	**0.63**
		**± SD**	**0.25**	**0.23**	**0.27**	**0.17**	**0.25**
	**Y-intercept**	**Mean**	**31.7**	**25.3**	**26.9**	**23.4**	**23.9**
		**± SD**	**29.7**	**8.2**	**9.3**	**8.4**	**6.7**
**Damage**	**Slope coefficient**	**Mean**	**0.60**	**0.66**	**0.54**	**0.77**	**0.62**
		**± SD**	**0.37**	**0.25**	**0.25**	**0.15**	**0.29**
	**Y-intercept**	**Mean**	**25.3**	**40.0**	**30.1**	**27.8**	**28.8**
		**± SD**	**6.5**	**19.1**	**14.4**	**8.2**	**7.3**

**Fig 5:**
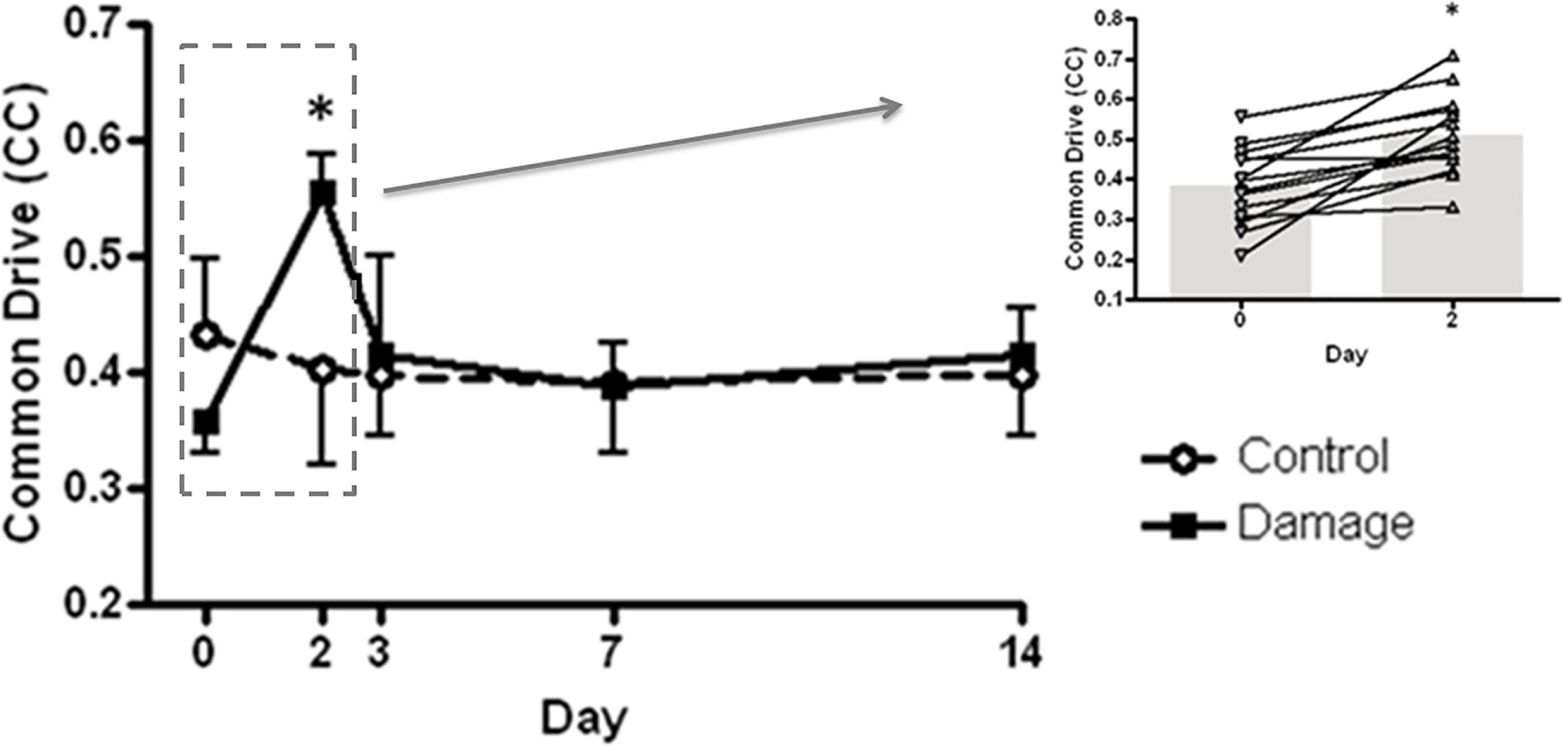
Cross correlation coefficient of active motor units in the exercised and control knee extensors. Values are mean + SD, n = 14. Cross correlation coefficients of active motor units for each individual participant between baseline (Day 0) and peak EIMD (Day 2) are presented inset. * Significantly higher than baseline in the exercised leg, p <0.05.

The mean number of motor units identified by PD III did not differ significantly (*F*_(4,13)_ = 0.62, p >0.05) across time or group, ranging from 19.8 ± 8.4–26.4 ± 9.1. Common drive, as shown by the cross correlation coefficient of active motor units, was significantly (*F*_(4,13)_ = 8.52, p <0.05) elevated from 0.36 ± 0.027 to 0.56 ± 0.032, with time lag of 0.05 ± 0.018ms and 1.78 ± 0.013ms respectively, and displayed significant interaction (*F*_(4,13)_ = 22.34, p <0.001) with the control leg, post hoc analysis revealed a significant increase 48h post-EIMD (CI [4.41 to 7.98] ES = 6.39, p <0.001) which returned to baseline after 72h (Fig 5).

## Discussion

This study aimed to determine how motor unit behaviour controlled force production during EIMD, and over the complete recovery period. Specifically, it was hypothesized that firing rates of high-threshold motor units would be most affected by EIMD, in line with impaired muscle function. Additionally, the collective control (common drive) of the motor unit pool was examined, before and after damaging exercise. EIMD was successfully induced as shown by the 31.4% MVC decline 48h post-exercise. This force decrease was accompanied by diminished rate of torque development and elevated muscle soreness up to 72h post-exercise. Taken together these findings are symptomatic of EIMD [7,64,68]. Coinciding with these functional impairments, the mean firing rate of high-threshold/ later recruited motor units declined by 22.3% 48h post-exercise. Recovery from the firing rate decline of these units occurred by 7 days post-EIMD, alongside MVC. The cross correlation coefficient or synchronization of the motor unit pool increased from 0.36 at baseline to 0.56 after 48h, indicating increased common drive.

As hypothesized we showed that later recruited motor units (i.e. higher-threshold) were specifically impaired following EIMD, as we know that later recruited motor units associate with type II muscle fibres [48] and that it is these fibres that are most susceptible to EIMD [35]. It seems likely that the observed impairment stems from a feedback mechanism resulting from elevated III/IV afferent signalling following EIMD [16,17]. Group III/IV afferents mediate inhibitory influence on motoneurones via several routes [69]; indeed, afferent feedback has been shown to impact upon planning of aimed movements, supraspinal cortical, subcortical and propriospinal motor outputs, as well as on the α-motoneurone at the spinal level, whilst also directly affecting motoneurone firing rates during muscle fatigue via reflex inhibition [69]. Mediation by III/IV afferents also appears a plausible explanation given the lack of any impairment in the contralateral limb [16]. Group III / IV afferents have previously been associated with reduced central motor drive, thereby inhibiting voluntary muscle activation, acutely during fatiguing exercise [70]. It is therefore possible that impaired MUFR may result from feedback from damaged muscle, designed to restrict function and thereby limit the risk of further harm.

Despite the decline in high-threshold motor unit firing rate, the 60% MVC target was successfully achieved during EIMD suggesting that some sort of compensation had occurred. We propose this came from the increased common drive we showed, which typically increases when higher forces are required [71] and during muscle fatigue [72]. However, Beck et al (2012) [39] surprisingly reported no alteration in common drive following EIMD in the biceps brachii, despite a 19.5% drop in peak force; it should be noted that the isometric contractions during which common drive was assessed were based on feedback provided by EMG (RMS) and not on a predetermined target force output, with this in mind, objective comparison cannot be made between pre- and post-exercise conditions. Nevertheless, the most likely mechanism causing the increased common drive in our study emanates from impaired proprioception which can occur following EIMD [73]. Indeed, Ye et al (2014) [37] reported increased common drive following eccentric, but not concentric, fatiguing exercise. It has been previously demonstrated [7,74] that EIMD transiently alters skeletal muscle architecture which is likely to alter proprioception from muscle spindles which has been suggested to influence common drive [75]. Furthermore, Contessa et al (2009) [72] observed a relationship between the number of newly recruited motor units and the common drive with contraction endurance time, leading them to propose a decreased muscle spindle influence would result in increased common drive. It seems that disruptions within the muscle could conceivably lead to a motor unit firing pattern which lends itself more towards greater force production, and less towards fine motor control.

Later recruited muscle fibres are preferentially damaged during EIMD [34,35], and in accordance with our hypothesis, we demonstrated that later recruited motor units (i.e. higher-threshold) were impaired following EIMD. This is the first study to demonstrate impairment and recovery of high-threshold motor unit firing rate, in association with recovery of MVC. Previous studies have demonstrated divergent time courses of neural and functional alterations, however ours is the first study to assess neural behaviour at an intensity of contraction (60% MVC) sufficiently high to recruit a broad representation of the motor unit pool, which we suggest provides fresh insight, using a functionally relevant stimulus. These findings suggest that altered neural firing occurs at relatively high, submaximal force levels, during periods of reduced muscle function, which we propose, may serve to protect damaged muscle throughout the acute recovery phase. This observation has important implications within populations at heightened risk of injury or impaired mobility.

This study provides new evidence that the acute insult, inflicted upon muscle through exercise-induced muscle damage, is associated with transient decreases in firing rate among later recruited motor units with higher recruitment thresholds. These decreases, and subsequent recovery, coincide with alterations in force production. Low-threshold units, recruited early after the onset of contraction, remain unaffected throughout. These findings suggest that changes in motor unit activity following exercise-induced muscle damage may mediate recovery of force following exercise-induced muscle damage.

## Supporting information

S1 Table Peak torque recorded during maximal voluntary contraction. (CSV)

S2 Table Rate of torque development recorded during maximal voluntary contraction. (CSV)

S3 Table Subjective rating of pain according to visual analogue scale. (CSV)

S4 Table Mean firing rate of early (T1), mid (T2), and later (T3) recruited motor units. (CSV)

S5 Table Cross correlation coefficient of active motor units. (CSV)
